# Transcriptomics and Metabolomics Identify Drug Resistance of Dormant Cell in Colorectal Cancer

**DOI:** 10.3389/fphar.2022.879751

**Published:** 2022-04-08

**Authors:** Lang Xie, Renli Huang, Hongyun Huang, Xiaoxia Liu, Jinlong Yu

**Affiliations:** ^1^ Department of General Surgery, Zhujiang Hospital, Southern Medical University, Guangzhou, China; ^2^ Guangdong Provincial Key Laboratory of Colorectal and Pelvic Floor Disease, The Sixth Affiliated Hospital (Guangdong Gastrointestinal and Anal Hospital), Sun Yat-sen University, Guangzhou, China

**Keywords:** tumor dormancy, metabolomics, cancer-associated fibroblasts, colorectal cancer, transcriptomics

## Abstract

**Background:** Tumor dormancy is an important way to develop drug resistance. This study aimed to identify the characteristics of colorectal cancer (CRC) cell dormancy.

**Methods:** Based on the CRC cohorts, a total of 1,044 CRC patients were included in this study, and divided into a dormant subgroup and proliferous subgroup. Non-negative matrix factorization (NMF) was used to distinguish the dormant subgroup of CRC *via* transcriptome data of cancer tissues. Gene Set Enrichment Analysis (GSEA) was used to explore the characteristics of dormant CRC. The characteristics were verified in the cell model, which was used to predict key factors driving CRC dormancy. Potential treatments for CRC dormancy were also examined.

**Results:** The dormant subgroup had a poor prognosis and was more likely to relapse. GSEA analysis showed two defining characteristics of the dormant subgroup, a difference in energy metabolism and synergistic effects of cancer-associated fibroblasts (CAFs), which were verified in a dormant cell model. Transcriptome and clinical data identified *LMOD1, MAB21L2*, and *ASPN* as important factors associated with cell dormancy and verified that erlotinib, and CB-839 were potential treatment options.

**Conclusion:** Dormant CRC is associated with high glutamine metabolism and synergizes with CAFs in 5-FU resistance, and the key effectors are LMOD1, MAB21L2, and ASPN. Austocystin D, erlotinib, and CB-839 may be useful for dormant CRC.

## Introduction

Colorectal cancer (CRC) is one of the most common malignant tumors worldwide (Siegel et al., 2020), and recurrence after surgery and chemotherapy is a leading factor of CRC-related deaths (Katona and Weiss, 2020). Recent studies have shown that CRC cells can enter a reversible dormant state leading to chemotherapy resistance (Rehman et al., 2021). In the dormant state, cancer cells regulate their cell cycle to enter a slow cycle mode (Basu et al., 2022). This allows the cells to survive in hostile environments such as hypoxia, effects of the immune system, and the effects of chemotherapy (Ju et al., 2020; Manjili et al., 2022).

In this study, we identified a dormant subgroup of CRC cells based on the transcriptome data of CRC patients. We then clarified two characteristics in the dormant subgroup of CRC cells, energy metabolism reprogramming and synergized with CAFs, and verified the results *in vitro*. Furthermore, we identified dormancy-related genes that regulated drug resistance of dormant CRC cells and predicated three drugs that may be effective against dormant CRC cells.

## Materials and Methods

### Patients and Samples

In this study, we included 459 patients identified in The Cancer Genome Atlas-Colon Adenocarcinoma (TCGA‐COAD) population (https://portal.gdc.cancer.gov/) and 585 patients in GSE39582 (Marisa et al., 2013) of the Gene Expression Omnibus (GEO, http://www.ncbi.nlm.nih.gov/geo/). Raw RNA-sequencing data counts (level 3) of patients in the TCGA-COAD cohort were downloaded from the TCGA database as recommended by guidelines and converted into transcripts per kilobase million for analysis. Raw CEL files (Affymetrix DNA microarray image analysis) of patients in GSE39582 were downloaded from the website mentioned above, and processed by “affy” and “simpleaffy” R packages (Irizarry et al., 2003).

### Identification of CRC Subgroups

The Gene Set Enrichment Analysis (GSEA) website (https://www.gsea-msigdb.org/gsea/msigdb/cards/GOBP_CELL_CYCLE_ARREST.html) was searched for gene sets associated with cell cycle regulation, and the intersections between these gene sets and the genes in the TCGA-COAD and GSE39582 were identified and selected for subsequent analysis. Genes of low median absolute deviation value (≤0.5) across all datasets were excluded.

A total of 223 genes associated with cell cycle arrest genes were identified. These genes were processed by non-negative matrix factorization (NMF) clustering (Possemato et al., 2011) using the R software NMF package (version 0.23.0). The maximum number of clusters for consistency analysis was 6, and the matrix was drawn 50 times. The consensus map function (CMF) of the NMF package was used for producing clustered heatmaps. Rank values where the magnitude of the cophenetic correlation coefficient began to fall were chosen as the optimal number of clusters (Brunet et al., 2004).

### Characterization of CRC Subgroups and Identification of Dormancy-Related Genes

The Limma package (version: 3.40.2) of R software was used to study the differential expression of mRNAs. The Log (fold-change) of all genes in datasets were used to perform GSEA analysis in webgestalt (http://www.webgestalt.org/) (Liao et al., 2019) for identification and characterization of CRC subgroups. An adjusted *p* value <0.05 and Log (fold-change) > 1 or Log (fold-change) < −1 were defined as the thresholds for identifying differentially expressed genes (DEGs) among CRC subgroups. The intersections of DEGs between the TCGA-COAD and GSE39582 were selected for prognostic analysis. The Kaplan-Meier method and log-rank test was used to compare survival between groups, and data were reported as *p* value, hazard ratio (HR), and 95% confidence interval (CI). Univariate Cox proportional hazards regression analysis was performed using the R survival and survminer packages (version 4.0.3). DEGs with a value of *p* < 0.05 were considered dormancy-related genes. Gene Set Cancer Analysis (GSCA, http://bioinfo.life.hust.edu.cn/GSCA/) (Liu et al., 2018) was used to examine correlations between dormancy-related genes and cell cycle pathway activity.

### Cell Culture and Dormant Cell Model Construction

The CRC cells lines LoVo and RKO were donated by Dr Xiaoxia Liu (Affiliated Sixth Hospital, Sun Yat-sen University, Guangzhou, China). The CRC cell lines HCT116 and DLD1 were obtained from the American Type Culture Collection (ATCC, United States). Cells were cultured in DMEM medium (Code: C11875500BT, Gibco, United States) with 10% fetal bovine serum (FBS) (Code: 10270-106, Gibco), 100 μg/ml streptomycin and 100 U/mL penicillin in a 5% CO2 atmosphere at 37°C. 5-Fluorouracil (5-FU) (Code: HY-90006, MCE, United States) (200 µM) was added to the cultures as a proxy for more than 4 months to select drug-resistant persister cells.

### Cell Cycle Analysis

LoVo persister cells (LoVo-P) and RKO persister cells (RKO-P) were collected on day 1 and day 10 after the adding 5-FU. Cells in each group (1×10^6^) were pelleted, harvested after being starved for 24 h, fixed with 75% cold ethanol overnight, and stained with propidium iodide (PI) using a PI kit (Beyotime Biotechnology, Shanghai, China). Flow cytometry and the cell cycle module of FlowJo™ software (version 7.0) detected the distributions of cell cycle phases.

### Primary Human Cancer-Associated Fibroblasts Line Generation

Specimens of high-grade CRC (T4Nx) were obtained from chemotherapy naïve patients (age range 18–70 years) undergoing surgery at the Sixth Affiliated Hospital of Sun Yat-sen University (SYSU), China. The study was approved by the Human Medical Ethics Committee of the Sixth Affiliated Hospital of Sun Yat-sen University, and informed consent was obtained from all patients before surgery.

Primary CAFs were isolated from tumor specimens as previously described (Ligorio et al., 2019; Peng et al., 2021). Briefly, tumor tissue was chopped with a sterile scalpel and then digested for 3 h at 37°C using collagenase Digestion Medium (DMEM, penicillin 100 U/mL, streptomycin 100 μg/ml, collagenase digestion 125 units/mg). Following tissue digestion, cells were plated under adherent conditions in Growth Medium (DMEM, PenStrep 1X, 10% FBS) and passaged regularly. CAFs grew by adherence with fibroblast-like morphology and had a strong capacity for proliferation.

### Metabolon-Based Energy Metabolism Detection

LoVo-P or LoVo-nP cells were collected, and metabolites were extracted and detected. Briefly, cells (5×10^6^) were pelleted, washed with cold PBS, and snap-frozen in liquid nitrogen. Cells were then ultrasonicated at 4°C for 20 min, the supernatant was collected (20 min at 14,000×*g*, 4°C), and then sent for Metabolon-Associated Energy Metabolism analysis (Applied Protein Technology, Shanghai, China).

The supernatants were dried in a vacuum centrifuge. The dried samples were dissolved in 100 μL acetonitrile/water (1:1, v/v), adequately vortexed, and centrifuged (14,000 rpm, 4°C, 15 min). The supernatants were collected for liquid chromatography-tandem mass spectrometry (LC-MS/MS) analysis.

### U-^13^C Glucose Labeling

The indicated cells (LoVo-P/nP and RKO-P/nP) cells were grown to 80-90% confluence in 10 cm cell culture dishes and then cultured in glucose-free DMEM (Thermo Fisher Scientific) supplemented with 4.5 g/L U-^13^C-glucose (Cambridge Isotope Laboratories, Andover, MA) and 10% FBS for 24 h. The medium was removed, and the cells were washed twice in the culture dish with 2 ml saline without disturbing cell attachment. Next, 500 µl of methanol was added to the cells to quench metabolic reactions, followed by an equal volume of water. The cells were then collected by scraping and placed in 2 ml Eppendorf tubes, and 500 µl chloroform was added to each tube. The cell extracts were vortexed at 4°C for 30 min. The samples were centrifuged at 14,000×*g* for 5 min at room temperature. For analysis of polar metabolites, the upper layer of the aqueous phase (700 µl) was transferred to a new tube for evaporation under airflow (N2 gas or vacuum concentrator, 3 h, 45°C). The dried metabolites were stored at −80°C until LC-MS/MS analysis. The LC/MS was performed at the Metabolic Innovation Center (MIC) of Sun Yat-Sen University.

### Cell Survival and Proliferation Analysis

The cell survival rate was assessed using a Cell Counting Kit-8 (CCK-8) (Dojindo Lab, Japan) assay according to the manufacturer’s instructions. CAFs were isolated from primary tumor samples, and then we collected conditioned medium (CAF-co CM) from each of the CRC cells/CAFs co-culture. Briefly, a total of 5×10^4^ persister or parental cells were placed into the upper chamber in 0.2 ml of complete DMEM, and 2×10^5^ CAF cells in 1 ml of complete DMEM was placed in the lower chamber, and then the cells were incubated for 24 h. The medium in the lower chamber was collected and defined as CM/co-P and CM/co-nP, respectively. The CM was filtered with a 0.8 mm filter to remove cell debris and then used. After that, a total of 5,000 cells/well were seeded into 96-well plates overnight, then cultured in DMEM containing 10% FBS with 25% CAF-co CM or control medium and treated with the indicated concentrations of 5-FU for 72 h. The absorbance at 450 nm was measured using a Thermo Scientific Varioskan Flash instrument after incubation with 10 µl CCK-8 solution for 2 h at 37°C, and the proliferation index was calculated.

Cell proliferation was also assessed via IncuCyte ZOOM (Essen BioScience). A total of 5,000 cells/well were cultured as described above 96-well plates and were automatically monitored and data recorded every 2 h by IncuCyte ZOOM for 72 h.

### Drug Sensitivity Analysis

Gene Set Cancer Analysis (GSCA, http://bioinfo.life.hust.edu.cn/GSCA/) was used for analyzing correlations between the expressions of dormancy-related genes and drug sensitivity of cell lines in the Cancer Therapeutics Response Portal database (CTRP, http://portals.broadinstitute.org/ctrp/). DMEM medium with CB‐839 (Code: S7655, Selleck, United States)] and erlotinib (Code: HY-50896, MCE, United States) (2 μM) was used for evaluating drug sensitivity rescue.

### Statistical Analysis

Statistical analysis was performed using R (version 4.0.2) and GraphPad Primer 8.0 (GraphPad Prism, GraphPad Software, La Jolla, CA). All *p* values were 2-tailed, and values <0.05 were considered to indicate statistical significance.

## Results

### Definition of CRC Dormancy or Proliferation

**FIGURE 1:**
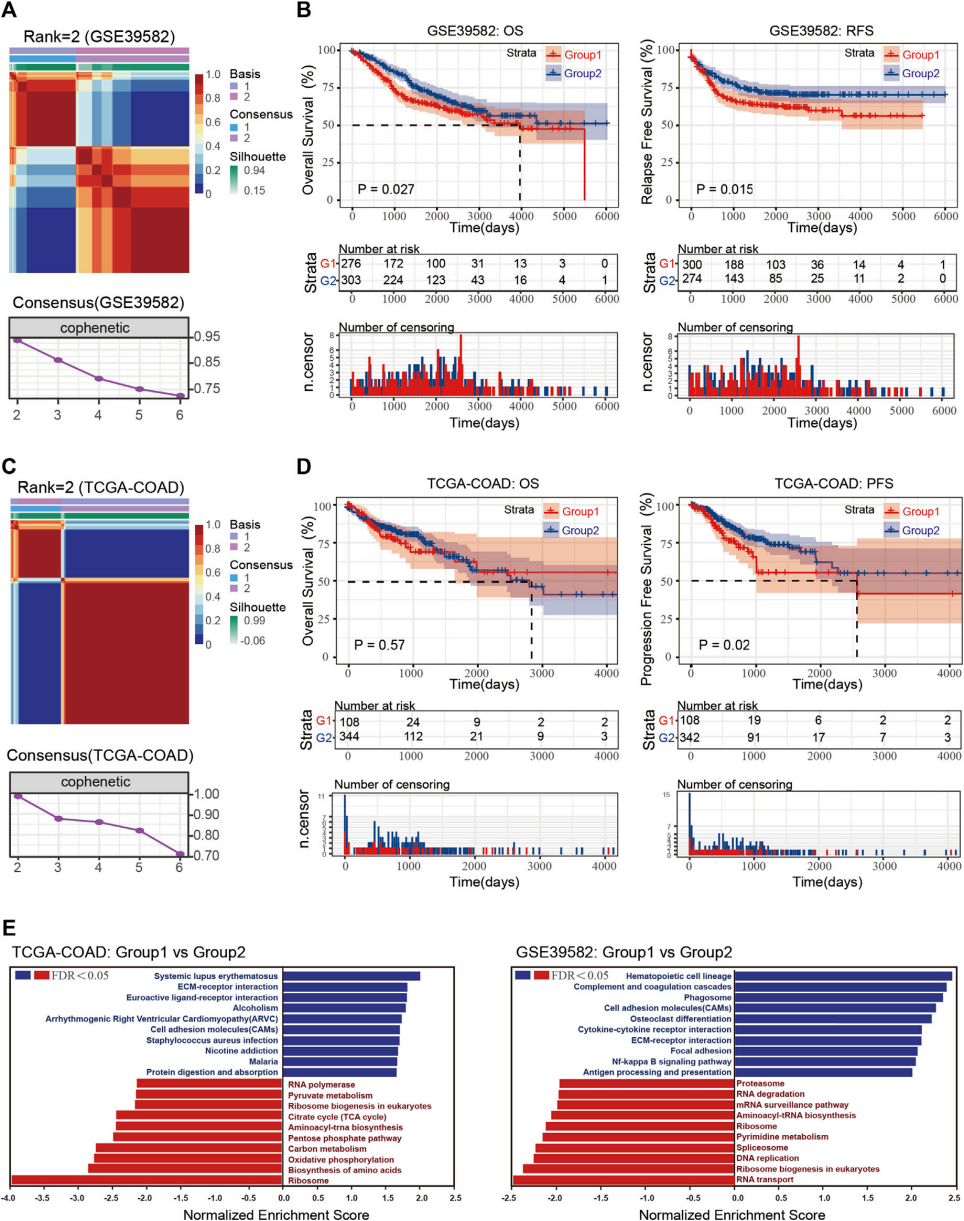
**(A)** Consensus map and consensus results of GSE39582 with rank = 2. **(B)** Overall survival (OS) and recurrence-free survival (RFS) of the dormant and proliferous subgroups in GSE39582. **(C)** Consensus map and consensus results of TCGA-COAD with rank = 2. **(D)** OS and RFA of the dormant and proliferous subgroups in TCGA-COAD. **(E)** GSEA results of Group 1 vs. Group 2 in TCGA-COAD and GSE39582.

To define dormancy or proliferation in CRC, we first selected gene sets in cell cycle arrest (GO: 0007050) and used their expression data for clustering by NMF. As shown in Figures 1A,C, following the rules of NMF, the optimum number of the cluster was two in both GSE39582 and TCGA-COAD gene sets. After defining the two groups, GSEA analysis was performed, and the normalized enrichment score (NES) and false discover rate (FDR) were determined; an FDR <0.25 was considered significant. Only the top and bottom categories (according to NES) are shown in Figure 1E. The results showed that hypoactive Kyoto Encyclopedia of Genes and Genomes (KEGG) pathways in the two datasets reflected processes of cell proliferation such as ribosome generation, DNA replication, and RNA synthesis. As shown in Figure 1, the proliferation ability of CRC in group 1 was weaker than that of group 2; thus, group 1 was considered the dormant subgroup and the other the proliferous subgroup.

Increasing evidence has shown that dormant cells can evade the effects of chemotherapy and cause cancer relapse. Thus, we examined prognostic data of the two groups. The dormant subgroup showed an unfavorable prognosis concerning overall survival (OS) and recurrence-free survival (RFS) in the GSE39582 dataset and an unfavorable prognosis for progression-free survival (PFS) in the TCGA-COAD dataset (Figures 1B,D). In addition, the GSEA results also indicated an enhanced interaction between cells and matrix in the dormant group of both datasets and obvious differences in energy metabolism in the TCGA-COAD dataset.

### Metabolic Reprogramming in Dormant Subgroup

**FIGURE 2:**
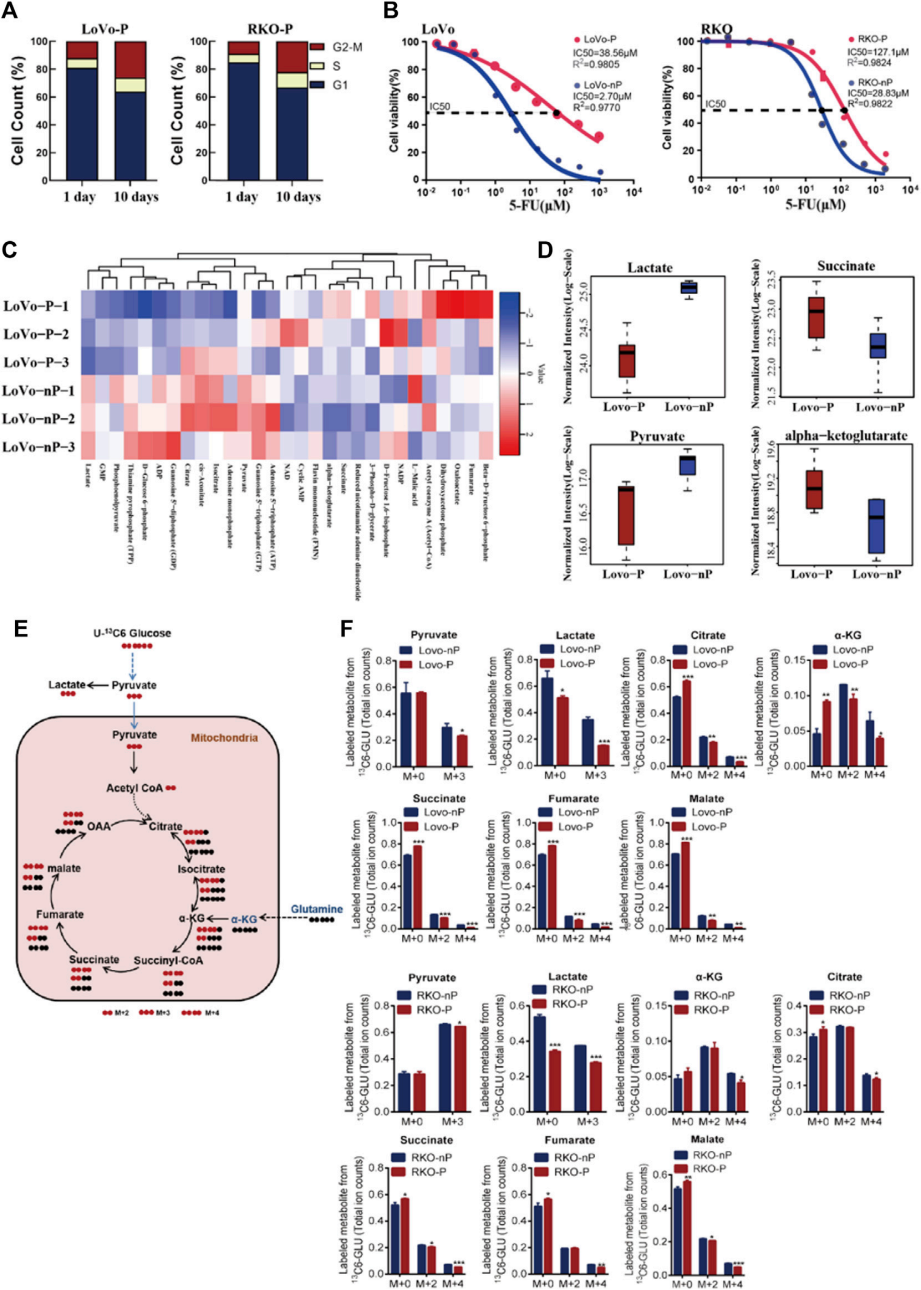
**(A)** Distribution of cell cycle phases in LoVo-P and RKO-P cells on day 1 and 10 after treatment with 5-FU. **(B)** Half maximal inhibitory concentration (IC50) of 5-FU between DTP cells (LoVo-P and RKO-P) and parental cells (LoVo-nP and RKO-nP). **(C)** Heatmap of metabolites in the energy pathway determined by metabolomics in LoVo-P and LoVo-nP cells. The levels of energy metabolites are shown in Supplementary Table S1. **(D)** Compared to LoVo-nP cells, the main discriminant metabolite levels in LoVo-P cells. **(E)** Schematic illustration of glucose recycling using U-^13^C-glucose. **(F)** U-^13^C-glucose tracing analysis of LoVo and RKO DTP cells and parental cells. Cells were cultured in U-^13^C-glucose and DMEM for 24 h to synthesize U-^13^C metabolites (*n* = 3). Bars, mean ± SD. *p < 0.05; **p < 0.01; ***p < 0.001.

In order to verify the characteristics of the dormant subgroup and clarify the differences between the two subgroups, we constructed a model of the dormant subgroup *in vitro* (Cho et al., 2021). CRC cell lines LoVo and RKO were used to construct persister cells by treatment with 5-FU. After the 5-FU induction period, the proliferation rates of persister cells (LoVo-P and RKO-P) were significantly reduced compared with the parental LoVo and RKO (LoVo-nP and RKO-nP) cells (Figure 2A), and they exhibited more resistance to 5-FU (Figure 2B). We then examined the cell cycles of persister cells at different times after removing 5-FU, and the results showed that LoVo-P and RKO-P cells could return to a proliferative state.

To better understand the metabolic changes associated with the dormant status, energy metabolism-related metabolites were examined by LC-MS/MS-based metabolomics (Figure 2C). A total of 29 out of 31 metabolites were identified to be energy metabolism-related, and there were significant differences in the levels between the two subgroups (Supplementary Table S1). A total of 12 different metabolites were significantly altered in persister cells. Compared with the parental group, five metabolites (*α*-KG, succinate, cyclic AMP, FMN, 3-phospho-d-glycerate) were increased, and seven metabolites (lactate, cis-aconitate, NAD, isocitrate, citrate, pyruvate, GTP) were decreased in the persister group. Overall, the results showed that the metabolite levels involved in glycolysis (lactate and pyruvate) were drastically reduced, whereas persister cells had increased levels of metabolites involved in glutaminolysis (such as *α*-KG and succinate). The results suggest that drug-tolerant persister (DTP) cells may have altered their metabolic requirements in response to the cytotoxic stress (Figure 2D).

Altered metabolism to sustain rapid growth is one of the hallmarks of cancer (Vander Heiden and DeBerardinis, 2017). Next, we further examined the metabolic changes in DTP cells by U-^13^C-glucose tracing and metabolomics analysis (Figure 2E). Consistent with our previous data, DTP cells showed a significantly lower enrichment in glycolytic ^13^C labeled lactate than did non-persister cells, indicative of the Warburg effect. By contrast, DTP cells had significantly higher fractions of *α*-KG (M+0), succinate (M+0), fumarate (M+0), malate (M+0), and citrate (M+0), indicating enhanced Krebs cycle and glutamine metabolism (Figure 2F). These data, along with the LC-MS/MS-based metabolomics data, indicate that DTP cells require glutamine metabolism to meet increased energy demands.

### Drivers of Dormant State: Dormancy-Related Genes and Synergistic Effect of Cancer-Associated Fibroblasts

**FIGURE 3:**
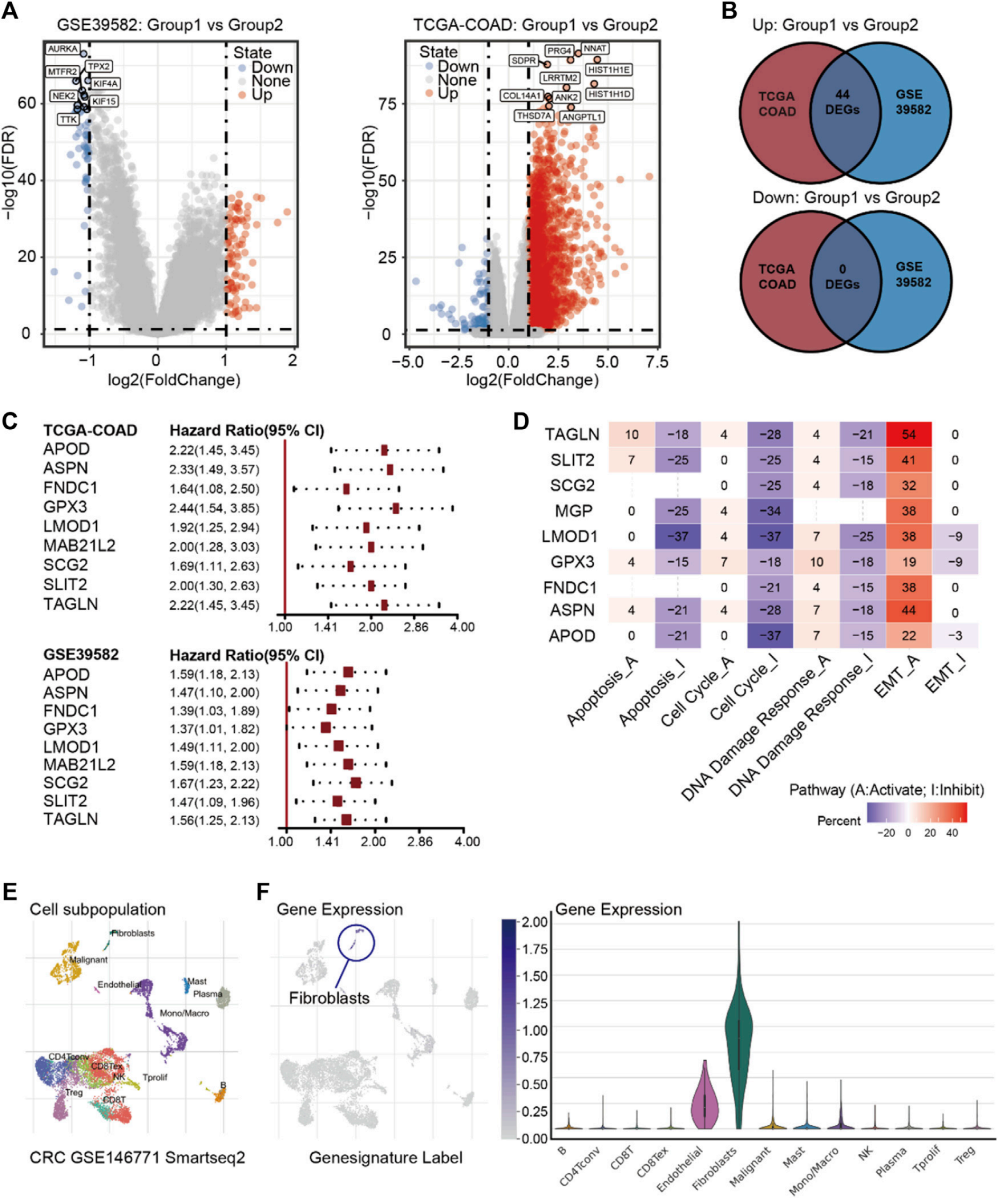
**(A)** Volcano plot of DEGs between Group 1 and Group 2 in GSE39582 and TCGA-COAD. **(B)** Intersections of DEGs between TCGA-COAD and GSE39582. **(C)** Cox proportional hazards regression analyses of nine prognosis-related genes in GSE39582 and TCGA-COAD. **(D)** Pathway activity of the nine prognosis-related genes. **(E)** Annotations of cell subpopulations in GSE146771. **(F)** Expressions of nine prognosis-related genes among cell subpopulations in GSE146771.

After confirming metabolic reprogramming in the dormant subgroup, we examined the enhanced interaction between cells and the extracellular matrix in the dormant subgroup. In order to clarify which stromal cells play critical roles, we first used TCGA-COAD and GSE39582 transcriptome data to determine DEGs between the two subgroups according to the pre-set conditions. As shown in Figures 3A,B, 44 up-regulated DEGs were identified as overlapping dormancy-related genes between TCGA-COAD and GSE39582 datasets. *RGS2* has been confirmed to regulate a dormant state in non-small cell lung cancer (NSCLC). Specifically, *RGS2* caused prolonged translational arrest in dormant cells through persistent eukaryotic initiation factor 2 phosphorylation *via* proteasome-mediated degradation of activating transcription factors (Cho et al., 2021). In addition, a cancer cell in a dormant state is always associated with chemotherapy resistance and tumor relapse; thus, we used Cox proportional hazards regression analyses to identify prognosis-related genes associated with RFS or PFS. The results showed that in both datasets, *APOD, ASPN, FNDC1, GPX3, LMOD1, MAB21L2, SCG2, SLIT2*, and *TAGLN* were potential genes causing or maintaining a dormant state of CRC (Figure 3C). Next, we used the GCSA database to explore the effects of these nine genes on common cancer-related pathways, and the results showed that they possibly negatively regulate the cell cycle and slow down cell metabolism (Figure 3D).

Next, we used the single-cell database GSE146771 (Zhang et al., 2020) and Characterizing Tumor Subpopulations (TISCH, https://http://tisch.comp-genomics.org//) (Sun et al., 2021) to ascertain the expressions of these nine genes in various stromal cells. The result showed that these nine genes are primarily highly expressed in fibroblasts (Figures 3E,F). While there is little evidence to show a direct correlation between fibroblasts and cancer dormancy, CAFs are the major subpopulation of fibroblasts in tumor stroma, and CAFs are also related to dormancy-related factors such as transforming growth factor-beta (TGF-*β*), interferon (IFN), insulin-like growth factor (IGF), fibroblast growth factor (FGF), macrophage-stimulating factor (M-SCF), and interleukin (IL) (Dai et al., 2021). This may indicate that CAFs can influence the state of cancer cells through remodeling the ECM, producing exosomes, mediating the balance of angiogenesis, or recruiting immune cells.

### CAFs Maintain the Drug-Tolerant Persister State of CRC Cells

**FIGURE 4:**
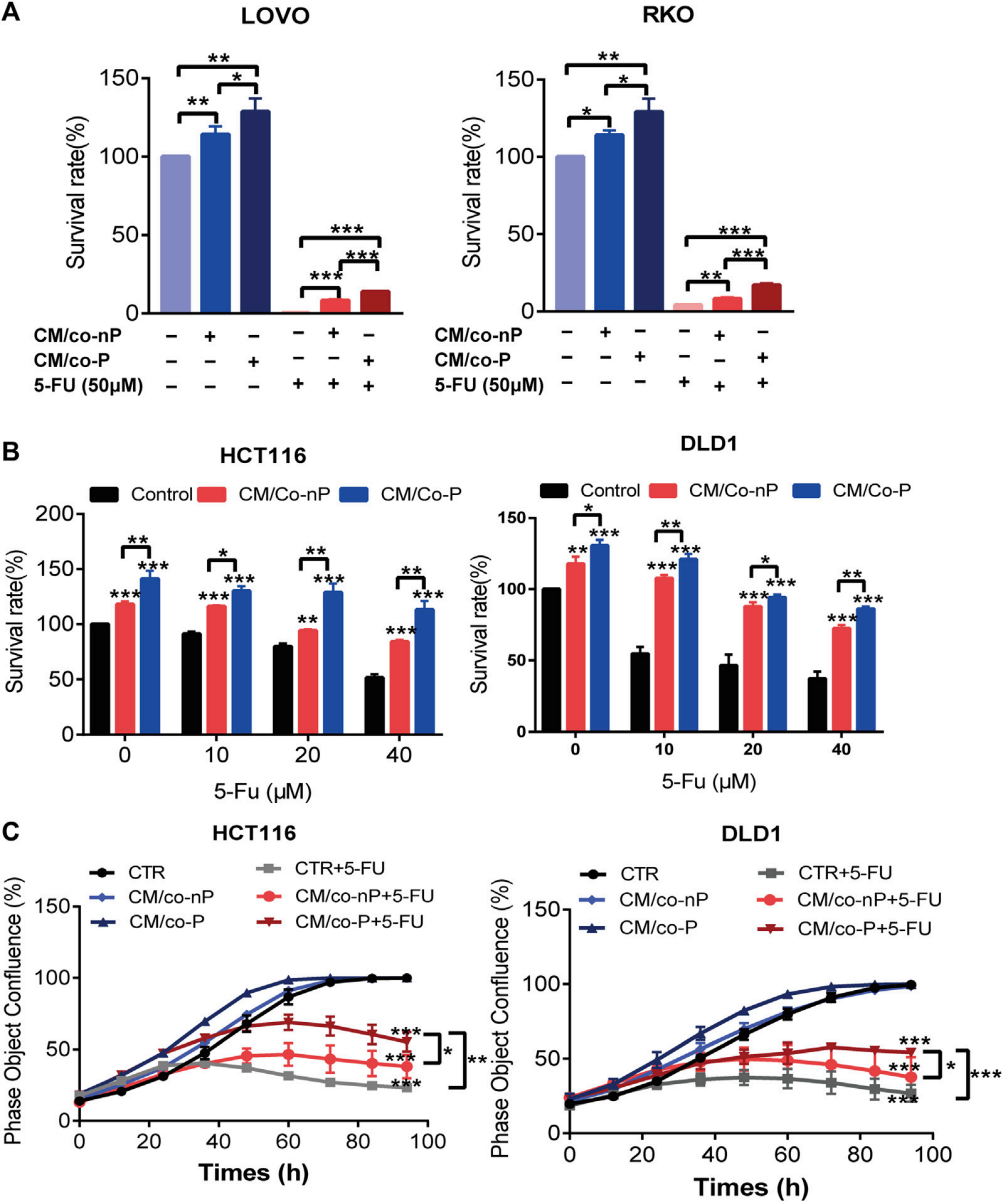
**(A)** Comparison of the sensitivity of LoVo (left panel) and RKO (right panel) cells to 5-FU after 72 h incubation with different CMs. Cell viability was measured by a CCK-8 assay. *n* = 3. **(B)** HCT116 (left panel) and DLD1 (right panel) cells incubation with indicated CM were treated with different concentrations of 5-FU for 72 h, and cell viability was measured by CCK-8 assay. *n* = 3. **(C)** Growth curves of HCT116 (left panel) and DLD1 (right panel) cells were cultured under the indicated conditions for 72 h. The image data for phase object confluence were processed by IncuCyte Zoom software. *n* = 3. Bars, mean ± SD. *p < 0.05; **p < 0.01; ***p < 0.001.

We speculated a synergistic effect between CAFs and CRC cells in maintaining the dormant state and enhancing drug resistance. In order to verify our hypothesis, the regular medium was replaced with CM/co-P or CM/co-nP and then treated with different concentrations of 5-FU for 72 h. The result showed that CM/co-P conferred more resistance to 5-FU in RKO and LoVo parental cells than CM/co-nP and control medium (Figure 4A). To further verify the efficacy of CM/co-P in other CRC cell lines, we used HCT116 and DLD1 cells. Consistently, the CCK-8 assay for cell survival showed that the CM/co-P also could induce 5-FU resistance in HCT116 and DLD1 cells even under a high concentration of 5-FU (40 μM) (Figure 4B). As shown in Figure 4C, Phase Object Confluence (%) detected by IncuCyte ZOOM further confirmed the chemotherapy-resistant in HCT116 and DLD1 cells after incubation with CM/co-P for up to 96 h treatment with 40 μM 5-FU. These data suggest that CM collected from CAFs following co-culture with DTP cells can protect against cytotoxic stress in CRC cells.

### Potential Treatment: Focus on *ASPN*, *LMOD1*, and MAB21L2 and Reverse the Drug-Tolerant Persister State of CRC

**FIGURE 5:**
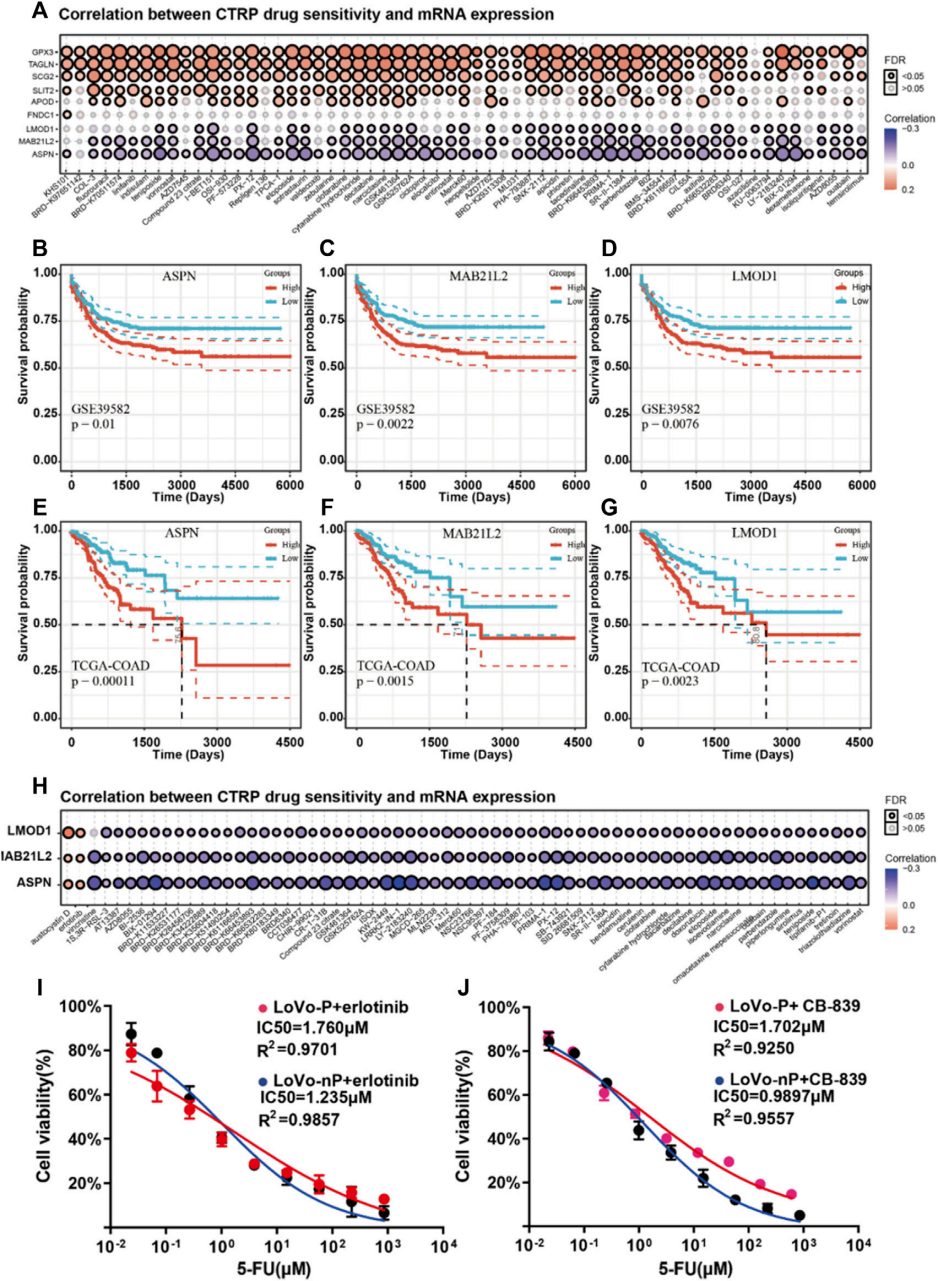
**(A)** Correlation analysis between mRNA expression of nine prognosis-related genes and CTRP drug sensitivity. **(B–D)** Recurrence-free survival (RFS) of *ASPN, LMOD1,* and *MAB21L2* in GSE39582. **(E–G)** Progression-free survival (PFS) of *ASPN, LMOD1,* and *MAB21L2* in GSE39582. **(H)** Correlation analysis between mRNA expression of *ASPN, LMOD1,* and *MAB21L2* and CTRP drug sensitivity. **(I-J)** IC50 of 5-FU between DTP cells (LoVo-P and RKO-P) and parental cells (LoVo-nP and RKO-nP) cultured with erlotinib (2 µM) and CB-839 (2 µM).

It has been shown that if tumor cells remain in a dormant state, they have developed significantly increased resistance to available chemotherapy drugs (Sosa et al., 2014). Following chemotherapy, residual DTP cells might re-enter the cell cycle and thus regain the ability to proliferate, causing a recurrence (Lin and Zhu, 2021). Therefore, it is critical to find new compounds to restore chemotherapy drug sensitivity that can kill DTP cells effectively. We used the CTRP drug database via GCSA (Liu et al., 2018) to analyze the correlations between expressions of the nine dormancy-related genes and drug sensitivity. All of the genes are up-regulated in the dormant subgroup, and the gene expression levels of *LMOD1, MAB21L2*, and *ASPN* increased significantly with drug resistance (Figure 5A). This result suggests that ASPN, LMOD1, and MAB21L2 are key proteins that drive chemotherapy resistance and tumor recurrence in the dormant subgroup, and CRC patients with high expression of *LMOD1, MAB21L2*, and *ASPN* are at high risk of recurrence (Figures 5B–G). In addition, using the CTRP drug database, we found that sensitivity to austocystin D and erlotinib increased as *LMOD1, MAB21L2*, and *ASPN* expression increased (Figure 5H).

Interestingly, the EGFR signaling pathway is related to tumor dormancy and drug resistance (Luo et al., 2018). Besides, The transport of glutamine is also related to the drug sensitivity of cetuximab (Ma et al., 2018). Therefore, we speculated that they might contribute to the death of DTP cells in CRC. Thus, we verified our hypothesis using DTP cells (LoVo-P, RKO-P), and the results suggested that erlotinib can restore chemotherapy drug sensitivity and kill DTP cells (Figure 5I).

Austocystin D is a newly developed anti-cancer drug and is reported to overcome chemoresistance (Marks et al., 2011). However, it was not available to determine its effect on DTP cells. What is more, we used CB-839 to inhibit cells’ glutamine metabolism and found that targeted GLS/GLS2 were the other strategies for overcoming chemoresistance of DTP cells (Figure 5J). Therefore, erlotinib, CB-839 may be potential treatments for overcoming resistance to chemotherapy in CRC patients.

## Discussion

Tumor dormancy is a significant factor in chemoresistance and cancer relapse. Identifying and characterizing tumor dormancy can help develop appropriate strategies for reversing the dormant state to overcome chemoresistance. However, nearly all of the research regarding the dormant state of cancer is based on cell and animal models. Our research began with transcriptome data from two large cohorts of patients with CRC and divided the patients into those with dormant and proliferous subgroups. GSEA analysis indicated that the two cohorts have strong consistency. It is generally believed that increased tumor proliferative activity in cancer patients often predicts poor outcomes, such as rapid progression and a poor prognosis, while weak proliferative activity is associated with a low degree of invasiveness and a good prognosis. In this study, patients in the dormant group were at high risk of cancer recurrence. Furthermore, based on the GSEA analysis, we identified two major characteristics of the dormant subgroup; metabolic changes and enhanced interactions with the cell matrix. At present, there are no other studies that have examined metabolic changes in dormant CRC cells. In this study, we found that indicators of cell energy metabolism such as pyruvate metabolism, the TCA cycle, and oxidative phosphorylation in the dormant subgroup were significantly different from that of the proliferous group, and we found that lactic acid and pyruvate in dormant cells were significantly reduced, which indicated the reversal of the Warburg effect (Vander Heiden et al., 2009).

Studies have shown that tumor cells can switch between aerobic glycolysis and oxidative phosphorylation to survive in a high-stress environment (Wangpaichitr et al., 2017). This adaptability is achieved by changing the method of energy metabolism, called metabolic reprogramming (Faubert et al., 2020). Metabolic reprogramming is a dominant evolutionary choice in tumor cells’ malignant transformation, which aids in survival (Herst et al., 2018). During chemotherapy, drug-resistant cells usually exhibit higher mitochondrial activity (Daniel et al., 2021). Drug-resistant cells may also rely on fatty acid metabolism or glutaminolysis to sustain their energy needs. Our results showed that the synthesis of lactic acid and pyruvate in dormant cancer cells were decreased compared with that of parent cells. We also found increased metabolites of the TCA cycle, such as *α*-KG and succinate, which indicates that energy reprogramming may be present, and there are alternate synthetic pathways of these metabolites in DTP cells. This phenomenon illustrates the importance of glutamine metabolism (Altman et al., 2016; Martinez-Outschoorn et al., 2017), which can synthesize *α*-ketoglutarate into the TCA cycle to produce ATP from glutamine *via* glutaminase and glutamate. Therefore, we detected the flow of carbon sources in dormant CRC cells and confirmed our hypothesis that dormant cells significantly increased the effect of glutamine metabolism. This finding indicates that glutamine is an energy source of dormant CRC cells. Interestingly, a recent study revealed that CRC cells exhibited the highest glutamine uptake in the tumor microenvironment, whereas myeloid cells had the greatest capacity to take up intra-tumoral glucose (cell-programmed nutrient partitioning) in the tumor microenvironment.

In addition, other GSEA analysis results suggested that the interaction between cells and matrix was enhanced in the dormant subgroup. Further analysis using the single-cell database indicated that fibroblasts play a critical role in the interaction. No prior studies examine the interaction between dormant CRC cells and cancer-associated fibroblasts; however, studies have shown that CAFs are closely related to tumor cell proliferation and survival. They can release lactate and glutamine to create a nutrient-rich microenvironment that assists tumor cell survival (Whitaker-Menezes et al., 2011; Kim et al., 2013). The most critical regulatory factor is hypoxia-inducible factor 1α (HIF-1*α*), as HIF-1*α* can regulate the expression of microcystin 1 (Cav-1) to affect the autophagy level of CAFs (Kannan et al., 2014). Studies have also shown that fibroblasts lacking Cav-1 secrete more glutamine into the microenvironment (Sotgia et al., 2012; Zhao et al., 2016). This mechanism is consistent with the metabolic characteristics of dormant CRC cells. i.e., increased demand for glutamine.

CAFs consume more glucose in most solid tumors and secretes more lactic acid than normal fibroblasts (Zhao et al., 2016). Our co-culture results showed that CAFs could enhance the drug resistance of DTP cells. The founding suggests that dormant CRC cells interact with CAFs to promote glutamine metabolism and resist the effects of chemotherapy. In order to determine the key drivers of this effect, we identified 44 DEGs in the two cohorts. Prior study has shown that *RGS2* is a driver of dormant cells in NSCLC (Cho et al., 2021). Subsequently, nine prognosis-related genes were identified through univariate analysis, and finally, *LMOD1, MAB21L2*, and *ASPN* were established as potential genes resulting in drug resistance in CRC dormancy via the CTPR database. We also predicted that erlotinib, CB-839, and austocystin D could potentially kill dormant CRC cells and verified the impact of erlotinib and CB-839 with DTP cells. According to the Human Protein Atlas (HPA, https://www.proteinatlas.org/), ASPN and MAB21L2 is located in the nucleus. In addition, studies have shown that ASPN is enriched in CAFs (Hesterberg et al., 2021) and can promote cancer cell metastasis via regulating cell metabolism (Sasaki et al., 2021).

Further studies of how these predicted driver genes regulate the dormant state of CRC are warranted. In subsequent research, we will focus on ASPN and regulating CRC dormancy. Establishing the mechanism of drug resistance in CRC dormant cells may assist in the development of new types of chemotherapy that can improve the survival of patients with CRC.

## Data Availability

The datasets presented in this study can be found in online repositories. The names of the repository/repositories and accession number(s) can be found in the article/Supplementary Material.

## Glossary

*α*-KG: alpha ketoglutarate
APOD: apolipoprotein D
ASPN: asporin
ATP: adenosine triphosphate
CAFs: cancer-associated fibroblasts
CM: conditioned media
Cav-1: microcystin 1
CHARTS: characterizing tumor subpopulations
CRC: colorectal cancer
CTRP: Cancer Therapeutics Response Portal database
DEGs: differentially expressed genes
DTP: drug-tolerant persister
EGFR: epidermal growth factor receptor
FMN1: formin 1
FNDC1: fibronectin type III domain containing 1
FGF: fibroblast growth factor
GSCA: Gene Set Cancer Analysis
GTP: guanosine triphosphate
GPX3: glutathione peroxidase 3
HIF-1α: hypoxia-inducible factor 1 alpha
HR: hazard ratio
IFN: interferon
IGF: insulin-like growth factor
IL: interleukin
LC-MS/MS: liquid chromatography-tandem mass spectrometry
LMOD1: leiomodin 1
MAB21L2: Mab-21 like 2
M-SCF: macrophage-stimulating factor
NAD: nicotinamide adenine dinucleotide
NES: Normalized Enrichment Score
NMF: non-negative matrix factorization
OS: overall survival
PFS: progression-free survival
RGS2: regulator of G protein signaling 2
RFS: recurrence-free survival
SCG2: secretogranin II
SLIT2: slit guidance ligand 2
TAGLN: transgelin
TCA cycle: tricarboxylic acid cycle
TCGA: The Cancer Genome Atlas
TGF-*β*: transforming growth factor beta
